# Winter Aconite (*Eranthis hyemalis*) Lectin as a cytotoxic effector in the lifecycle of *Caenorhabditis elegans*

**DOI:** 10.7717/peerj.1206

**Published:** 2015-08-20

**Authors:** Marie-Therese McConnell, David R. Lisgarten, Lee J. Byrne, Simon C. Harvey, Emilia Bertolo

**Affiliations:** Biomolecular Research Group, School of Human and Life Sciences, Canterbury Christ Church University, Canterbury, Kent, UK

**Keywords:** N-acetyl-D-galactosamine, Winter Aconite Lectin, *Caenorhabditis elegans*, Dauer larvae, Ribosome Inactivating Protein

## Abstract

The lectin found in the tubers of the Winter Aconite (*Eranthis hyemalis*) plant is an N-acetyl-D-galactosamine specific Type II Ribosome Inactivating Protein (RIP); Type II RIPs have shown anti-cancer properties, and hence have potential as therapeutic agents. Here we present a modified protocol for the extraction and purification of the *E. hyemalis* lectin (EHL) using affinity chromatography. *De novo* amino acid sequencing of EHL confirms its classification as a Type II Ribosome Inactivating Protein. The biocidal properties of EHL have been investigated against the nematode *Caenorhabditis elegans*. Arrested first stage larvae treated with EHL have shown some direct mortality, with surviving larvae subsequently showing a range of phenotypes including food avoidance, reduced fecundity, developmental delay and constitutive dauer larvae formation. Both inappropriate dauer larvae development and failure to locate to bacterial food source are consistent with the disruption of chemosensory function and the ablation of amphid neurons. Further investigation indicates that mutations that disrupt normal amphid formation can block the EHL-induced dauer larvae formation. In combination, these phenotypes indicate that EHL is cytotoxic and suggest a cell specific activity against the amphid neurons of *C. elegans*.

## Introduction

Lectins are a class of carbohydrate binding proteins ubiquitously expressed in plants, animals, bacteria and viruses, characterised by their ability to agglutinate erythrocytes (Peumans & Van Damme, 1995), a property that enabled the development of the ABO system of blood typing. The second characteristic common to all lectins is the ability to bind carbohydrates selectively based on the individual sugar specifity of the lectin. This also results in lectins binding to the carbohydrate moieties of extracellular glycoconjugates specifically and reversibly without introducing conformational changes to the mono- or oligosaccharides to which they bind (Sharon & Lis, 2004). Plant lectins are involved in a wide range of processes including carbohydrate transport, cell–cell signalling, cell surface binding and recognition, pathogenic defence and in potentially mediating symbiotic relationships (Sharon & Lis, 2004). Plant lectins play a key role in defence, with many specifically binding to epithelial cells of herbivore and nematode guts (Schubert et al., 2012; Delatorre et al., 2007). Insecticidal, antifungal and antiviral qualities have also been widely described (e.g., Kumar et al., 1993; Rao et al., 1998; Peumans, Hao & Van Damme, 2001; Edwards & Gatehouse, 2007). For example, balsamin, from *Momordica balsamina*, demonstrates potent anti-HIV activity (Kaur et al., 2013).

In recent years the potential of lectins for use in cancer therapies has become a significant research focus due to their ability to preferentially bind to specific carbohydrates, and differentiate between glycosylation patterns. Moreover, a number of plant derived lectins have been shown to have potent *in vitro* and *in vivo* anti-cancer effects (e.g., Voss et al., 2006; Otsuka et al., 2014) inducing autophagous and apoptotic pathways in tumour cells, and some are already used therapeutically. For instance, the recombinant mistletoe lectin rViscumin has been through phase 1 clinical trials and a number of other native mistletoe lectin preparations such as Lektinol and Iscador are prescribed widely throughout Europe as adjuvant therapies although their efficacy is not readily quantified (Horneber et al., 2008).

Using a modified extraction protocol developed from previously published studies (Cammue, Peeters & Peumans, 1985; Kumar et al., 1993; George et al., 2011), this paper focuses on the lectin found in the tubers of Winter Aconite, *Eranthis hyemalis*, (EHL). To date, EHL is the sole lectin representative described from the *Ranunculaceae* and has been identified as a Type II Ribosome Inactivating Protein (RIP) (Kumar et al., 1993). EHL preferentially binds N-acetyl-galactosamine, but also binds galactose, galacto-pyransosyl-D-glucose, and to a lesser degree D-ribose (Kumar et al., 1993). Type II RIPs are classified as chimerolectins and cytotoxic N-glycosidases and consist of either one or two heterodimers linked by disulphide bonds. The B chain subunit is a sugar specific lectin containing the highly conserved ricin B domain and will bind to extracellular glyconjugates. This mediates entry to the intracellular environment for the attached cytotoxic A chain by endocytosis (Virgilio et al., 2010). The toxin is then subjected to retrograde transport via the Golgi complex; to the endoplasmic reticulum where the disulphide bonds are reduced and the A chain is free in the cytosol to refold into an enzymatically active form (Hartley & Lord, 2004). The A chain acts as an inhibitor of eukaryotic protein synthesis by cleaving a single adenosine (A_4234_) in the 28s rRNA subunit preventing Elongation Factor 2 from binding and resulting in immediate and absolute cessation of peptide elongation (Hartley & Lord, 2004).

Type II RIPs are an area of increasing interest due to their antineoplastic properties, and their glycomic binding profile can be used to target specific glycans of biological molecules. For instance, the GalNac specific RIP *Ximenia americana* (Riproximin) (Voss et al., 2006), *Sambucas sp* (Ferreras et al., 2011) and ML1 from *Viscum album* (Tonevitsky et al., 1996) show higher binding affinity for tumour cells than for healthy cells. This can be partly attributed to the expression of particular surface saccharide groups in the changing glycomics of malignant cells (Peumans, Hao & Van Damme, 2001; Bayer et al., 2012). The use of lectins and Type II RIPs is also indicated in some studies for marking metastatic proliferation of tumour cells due to excessive glycosylation in metastatic cell lines (Zhou et al., 2015).

Herein we present a modified extraction and purification protocol for EHL. Protein sequencing of EHL further supports the findings of earlier work that EHL is a Type II RIP. We also investigate the effect of EHL on the free living nematode *Caenorhabditis elegans*. *C. elegans* is a well-established model organism for initial toxicological studies due to the conserved nature of its biological and biochemical processes including stress response and disease pathways (Boyd, Smith & Freedman, 2012). Our research has revealed a range of phenotypes including direct mortality and a constitutive dauer formation phenotype that is consistent with neuronal ablation.

## Material and Methods

### Preparation of affinity chromatography column

An Amersham chromatography column was packed at room temperature with a final bed volume of 8 ml of Fetuin-Agarose in solution with 0.5 M NaCl and immobilised on cross-linked 4% beaded agarose (Sigma-Aldrich Company Ltd, UK). Prior to use the column was equilibrated with 8 column volumes of phosphate buffered saline (PBS).

### Extraction of EHL

60 g of *E. hyemalis* tubers supplied by Eurobulbs Ltd (UK) were prepared using a modified method from those described in previous studies (Cammue, Peeters & Peumans, 1985; Kumar et al., 1993; George et al., 2011) with adaptions as follows. The tubers were finely sliced before being homogenised with 250 ml of ice cold PBS containing 5 mM thiourea and left to settle on ice for 30 min. The homogenate was removed and stored and the remaining slurry was mixed with a further 250 ml of PBS. The two fractions were then combined and stirred at 4 °C for 4 h. The homogenised mixture was then centrifuged (Sorvall RC6 plus HSC) at 20,000 g for 30 min. The supernatant was retained and frozen at −80 °C overnight in order to induce aggregation of any remaining lipid content in sample. The sample was then defrosted and filtered through 3MM Whatman filter paper before undergoing a further centrifuge cycle of 20,000 g for 20 min. The clarified supernatant then underwent ammonium sulphate precipitation.

### Ammonium sulphate precipitation

Solid ammonium sulphate was added slowly to the crude extract initially to a saturation point of 40%, and after one hour of stirring at 4 °C was centrifuged at 10,000 g for 15 min. The pellet was re-suspended in 15 mls of PBS. Ammonium sulphate was then added to the supernatant to a final saturation point of 80%, with a further hour of stirring at 4 °C. The resulting pellet was also re-suspended in PBS. Agglutination activity was found to be retained in the 40% pellet and absent from the 80% pellet. An SDS-PAGE gel confirmed the presence of target protein in the 40% fraction. Samples were dialysed against PBS in 3 buffer changes consisting of 200× sample volume each including a final overnight exchange.

### Purification of EHL

The crude dialysed extract was applied to a fetuin-agarose affinity chromatography column at a rate of 1 ml per minute using ÄKTA Express protein purification system (GE Healthcare), non-target proteins were then eluted with PBS until absorbance at *λ*_280_ was restored to base line value circa 40 mAU. The affinity matrix was then equilibrated with PBS and subsequently EHL was eluted with 40 mM 1,3 diaminopropanol (DAP) and peak fractions were collected in 0.5 ml aliquots. The oligosaccharide structure of fetuin has been well defined and shown to have Gal and GlcNAc branched residues present. Fetuin has been purified using the lectin RCA I which has specificity for galactose and N-acetylgalactosamine (Green et al., 1988). Its use, therefore as an affinity chromatography media for the lectin/type II RIP purification is based on this complementary interaction. The eluant was neutralised with 2-Amino-2-hydroxymethyl-propane-1,3-diol hydrochloride (Tris-HCl) at pH 7.0. Peak fractions were applied to pre-equilibriated PD-10 desalting columns (GE Healthcare) and buffer exchanged into PBS.

### Analysis of EHL

Purified EHL was tested for agglutination ability using defibrinated rabbit erythrocytes (TCS Biosciences), with 20 µl of post column eluant, or control, added to a 20 µl sample of erythrocytes in a welled microscope slide. The purified EHL was also analysed by SDS-PAGE, with both reduced and non-reduced samples electrophoresed on 12% gels and subsequently stained with Coomassie Brilliant Blue. Concentration was measured using a Qubit flourometric protein assay.

Purified EHL was commercially sequenced. In-gel tryptic digestion was performed after reduction with DTE and S-carbamidomethylation with iodoacetamide. Gel pieces were washed two times with 50% (v:v) aqueous acetonitrile containing 25 mM ammonium bicarbonate, then once with acetonitrile and dried in a vacuum concentrator for 20 min. Sequencing-grade, modified porcine trypsin (Promega) was dissolved in the 50 mM acetic acid supplied by the manufacturer, then diluted 5-fold with 25 mM ammonium bicarbonate to give a final trypsin concentration of 0.02 µg/µL. Gel pieces were rehydrated by adding 10 µL of trypsin solution, and after 10 min enough 25 mM ammonium bicarbonate solution was added to cover the gel pieces. Digests were incubated overnight at 37 °C.

A 1 µL aliquot of each peptide mixture was applied to a ground steel MALDI target plate, followed immediately by an equal volume of a freshly-prepared 5 mg/mL solution of 4-hydroxy-*α*-cyano-cinnamic acid (Sigma) in 50% aqueous (v:v) acetonitrile containing 0.1%, trifluoroacetic acid (v:v).

Positive-ion MALDI mass spectra were obtained using a Bruker ultraflex III in reflectron mode, equipped with a Nd:YAG smart beam laser. MS spectra were acquired over a range of 800–5,000 m/z. Final mass spectra were externally calibrated against an adjacent spot containing 6 peptides (des-Arg^1^-Bradykinin, 904.681; Angiotensin I, 1,296.685; Glu^1^-Fibrinopeptide B, 1,750.677; ACTH (1–17 clip), 2,093.086; ACTH (18–39 clip), 2,465.198; ACTH (7–38 clip), 3,657.929). Monoisotopic masses were obtained using a SNAP averagine algorithm (C 4.9384, N 1.3577, O 1.4773, S 0.0417, H 7.7583) and a S/N threshold of 2.

For each spot the ten strongest precursors, with a S/N greater than 30, were selected for MS/MS fragmentation. Fragmentation was performed in LIFT mode without the introduction of a collision gas. The default calibration was used for MS/MS spectra, which were baseline-subtracted and smoothed (Savitsky-Golay, width 0.15 m/z, cycles 4); monoisotopic peak detection used a SNAP averagine algorithm (C 4.9384, N 1.3577, O 1.4773, S 0.0417, H 7.7583) with a minimum S/N of 6. Bruker flexAnalysis software (version 3.3) was used to perform spectral processing and peak list generation.

*De novo* sequencing of tandem mass spectra was performed by hand, with *a*-, *b*-, *b*^0^-, *y*-, *y*^0^- and *y*^∗^-ions considered as possible fragment ions. *De novo* derived peptides sequences were matched to homologous protein sequences using the on-line MS-BLAST service provided by Washington University. The results of which were consistent with the in-house homology search results conducted on confidently assigned sequences using the University of Virginia UVa FASTA Server, the FASTS and SSearch algorithms were used for homology searching against the SwissProt (NCBI) and PDB databases.

### Activity against *C. elegans*

Worms were obtained from the *Caenorhabditis* Genetics Center and maintained using standard methods (Stiernagle, 2006), on nematode growth media plates (NGM) using *Escherichia coli* OP50 strain food source. N2 was used for initial testing and as a control in other assays. To assess the effect of various mutations on EHL-induced dauer larvae formation, the following strains were used: CX2065, *odr-1(n1936)*; CX2205 *odr-3(n2150)*; PR671, *tax-2(p671)*; PR672, *che-1(p672)*; PR813,*osm-5(p813)*; SP1205, *dyf-1(mn335)*; and SP1709, *dyf-10(e1383)*. In all experiments, treatments and genotypes were blind coded, the position of plates within experimental blocks was randomised, and contaminated plates excluded from all analysis.

For all assays, arrested and synchronised *C. elegans* first stage larvae (L1s) were obtained by allowing eggs, isolated from gravid hermaphrodites by hypochlorite treatment (Stiernagle, 2006), to hatch on NGM plates in the absence of food for 24 h at 20 °C. For experiment 1, arrested N2 L1s were washed from plates, resuspended in M9 with a series of EHL concentrations from 3.92 to 0 mg/ml, incubated at 20 °C for 6 h, and 15 worms per treatment were picked for analysis. For experiment 2, arrested N2 L1s treated as above except treatments were 2.94 mg/ml and 0 mg/ml EHL and a greater number of worms per treatment were analysed (*n* = 55 and *n* = 33 for the 2.94 and 0 mg/ml treatments, respectively). After incubation, worms were washed 3 times in water. For the analysis of development and fecundity (experiments 1 and 2), worms were transferred in a small volume of liquid to NGM plates without food, then individually transferred from this plate to NGM plates with *E. coli* OP50 strain food source and maintained at 20 °C. Standard methods were then used to analyse the reproductive schedule and lifetime fecundity (Hodgkin & Doniach, 1997). These data were then used to assess the effect of EHL on reproduction as assessed by lifetime reproductive success (LRS), the total number of progeny produced (experiments 1 and 2), and the intrinsic rate of increase (*r*)(experiment 2), calculated by iteration from Σ*e*^−*rx*^*l_x_m_x_* = 1, where *l_x_* represents the age specific survivorship to day *x* and *m_x_* represents the fecundity on day *x* (Vassilieva & Lynch, 1999).

Based on phenotypes observed in the initial screen, the ability of EHL to induce constitutive dauer larvae formation (a dauer-constitutive, or Daf-c, phenotype) was investigated in greater detail. Here, worms were treated with 0.98, 1.96 or 2.94 mg/ml EHL, as described above, except that after washing, worms were transferred *en masse* to plates with food. After four days at 20 °C plates were visually scored to assess the proportion of worms that had developed as dauer larvae (number of dauer larvae/total number of worms). After counting, worms were washed from plates and incubated in 1% SDS for one hour, a treatment that kills all *C. elegans* stages except dauer larvae (Cassada & Russell, 1975), worms were washed once in M9, transferred to fresh NGM plates with food and the number of dauer larvae again counted. These dauer larvae were then transferred individually to NGM plates with food at 20 °C and monitored for the next 14 days to determine if they were capable of resuming development. To further analyse EHL-induced dauer larvae formation, N2 and mutant worms were treated, as described above, with 0, or 1.54 mg/ml EHL, washed, and transferred *en masse* to plates (*n* = 3 per combination of treatment and genotype) with food. Plates were then incubated at 20 °C for four days at which time the proportion of worms that had developed as dauer larvae was scored.

Dauer larvae formation in PR672, *che-1(p672)*, was further analysed both in standard dauer larvae formation assays and in assays of growing populations. For assays of dauer larvae formation in response to defined amounts of pheromone, assays were performed as previously described (Golden & Riddle, 1984; Green et al., 2014), with worms allowed to lay eggs on assay plates containing dauer pheromone extract and limited amounts of food, and progeny scored after two days at 25 °C. Dauer larvae formation in growing populations was assessed as previously described (Green et al., 2013), with populations initiated with single worms and a defined amount of food allowed to grow to food exhaustion, except that assays were performed at 25 °C.

## Results

### Purification and characterisation of EHL

**Figure 1:**
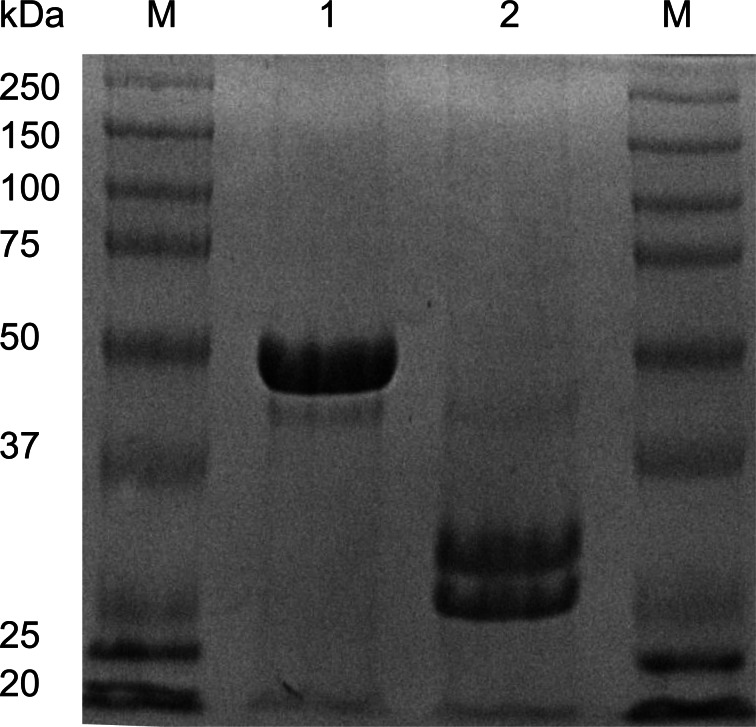
SDS-PAGE analysis of EHL. EHL is a heterodimeric Type II Ribosome Inactivating Protein. Coomassie Blue stained 12% SDS-PAGE analysis of EHL showing molecular weight marker (M), non-reduced EHL (1) and *β*-mercaptoethanol reduced EHL (2).

**Figure 2:**
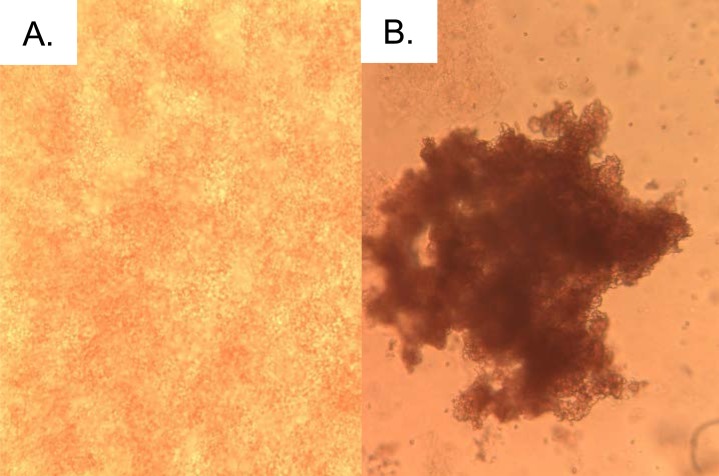
EHL induces agglutination of erythrocytes. (A) Control, with no clumping of erythrocytes observed, and (B) incubation with EHL, which results in agglutination.

Qubit fluorometric measurement showed a typical concentration of 2.5 mg/ml using our revised and improved purification strategy. This shows an approximately 5 to 6-fold increase in recovery in comparison to the previously reported yield of 380 µg/ml in George et al. (2011). Non-reducing SDS-PAGE analysis produced characteristic reduced protein bands at circa 31 and 28 kDa as well as an unreduced band circa 50 kDa (Fig. 1); these values are consistent with those previously reported in the literature (Kumar et al., 1993). An intense agglutination response of rabbit erythrocytes was exhibited and thus confirmed the presence of EHL (Fig. 2).

### De novo sequencing analysis

**Figure 3:**
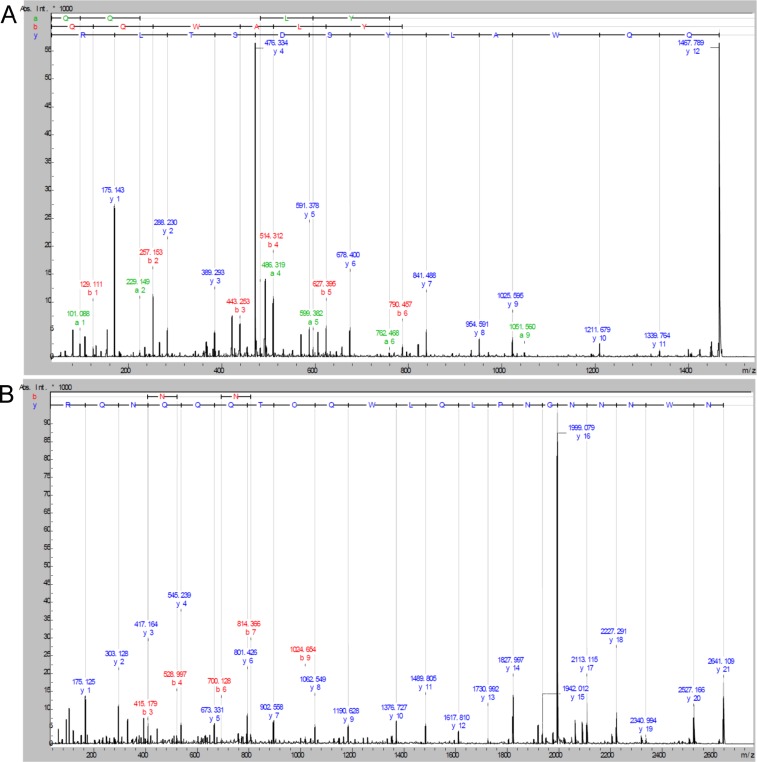
*De novo* peptide sequencing based on the MALDI-TOF MS/MS fragmentation spectra of EHL in-gel tryptic digestion after reduction with DTE and S-carbamidomethylation with iodocetamide. (A) QQWA(L/I)YSDST(L/I)R, the most confidently assigned peptide with a good level of overlapping *y-* and *b-*ion series. (B) NWNNNGNP(L/I)Q(L/I)WQCTQQQNQR, a strong *y-*ion series is observed throughout the peptide.

De novo sequence analysis of fragmentation spectra was carried out and suggested two confidently assigned peptide fragment sequences, with the following tags, QQWA[L/I]YSDST[L/I]R and NWNNNGNP[L/I]Q[L/I]WQCTQQQNQR (Fig. 3). Peptide fragment homology searches produced matches to various Type II Ribosome Inactivating Proteins (RIPS) all within ricin-b domain (carbohydrate binding) regions. This result confirms the status of EHL as a Type II RIP, as has been reported in previously published sequence data (Kumar et al., 1993). Sequence tags QQWA[L/I]YSDST[L/I]R and NWNNNGNP[L/I]Q[L/I]WQCTQQQNQR were matched to two regions in Nigrin-b (SNA-V; *Sambucas nigra agglutinin-V*) UniProtKB P33183.2 correlating to residues 470–481 and 325–345 respectively using the FASTS search facility against the SwissProt database. Close homology to other Type II RIPS was also matched within the ricin b domain of Abrin-a (P11140) and Ricin (P02879). PDB structures were searched using SSearch algorithm and also showed matches to SNA-II (3C9Z) (*Sambucas nigra* agglutinin II), Abrin-a (1ABRB) and Ricin (1RZOB) in the ricin b domain as well as ML1 from *Viscum album* (1QNKB).

### EHL affects survival, development and reproduction in *C. elegans*

**Figure 4:**
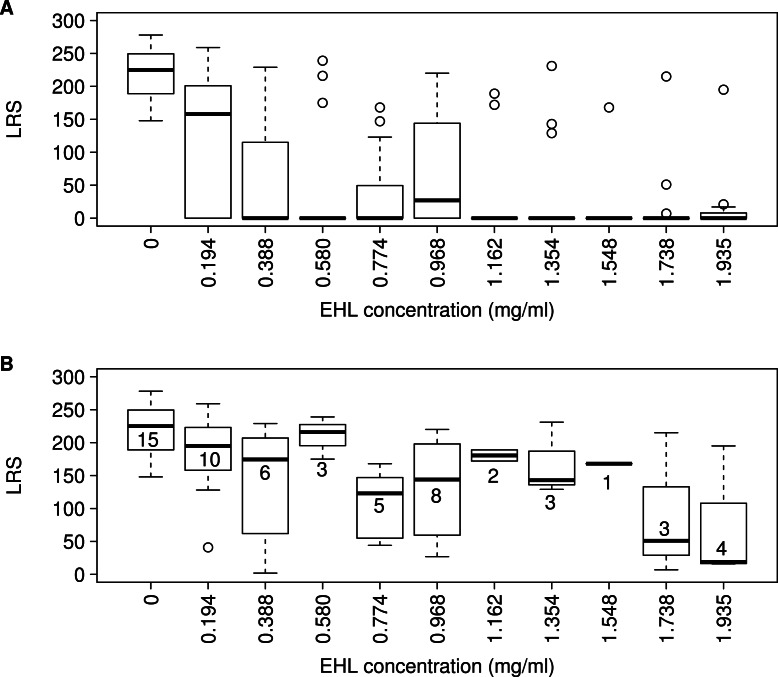
EHL reduces fecundity in *C. elegans*. Box plots of Lifetime reproductive success (LRS) of (A) EHL treated worms and (B) the subset of EHL treated worms that reproduced for a range of lectin concentrations. For (A) *n* = 15 for all treatments, for (B) numbers associated with the boxes denote the sample sizes.

Acute treatment of arrested *C. elegans* L1s for 6 h with EHL at different concentrations resulted in a subsequent range of developmental, fertility and survival defects (Fig. 4), with all EHL concentrations reducing lifetime reproductive success (LRS) (Fig. 4A, pairwise, Bonferroni corrected, Mann–Whitney U tests against N2 showing reduced fecundity in all EHL treatments). Much of this decrease in fecundity is however a consequence of worms not reproducing in the EHL treatments (comparison of Fig. 4A and 4B), although LRS does still decrease over the range of EHL concentrations tested when only those worms that reproduced are considered (Fig. 4B, Pearson product-moment correlation of LRS against EHL concentration: including the 0 mg/ml group, *r* = − 0.52, *p* < 0.001; excluding the 0 mg/ml group, *r* = − 0.38, *p* = 0.013). EHL-treated worms that did reproduce showed a delay in development, with many treated worms starting reproduction a day or more after the control worms. Of the worms that did not reproduce, some showed no movement from the point at which they were placed on the plate and no response to stimulus after 24 h and therefore died as a consequence of the EHL treatment. Other EHL-treated worms were observed to remain as arrested L1s or to develop as dauer larvae, a non-feeding developmentally arrested stage. Many of the non-reproducing worms were found not on a food source, and more than would be expected under these conditions were found to have climbed the sides of the plate; both behaviours are indicative of a disruption to chemosensory ability and an inability to detect the bacterial food.

**Figure 5:**
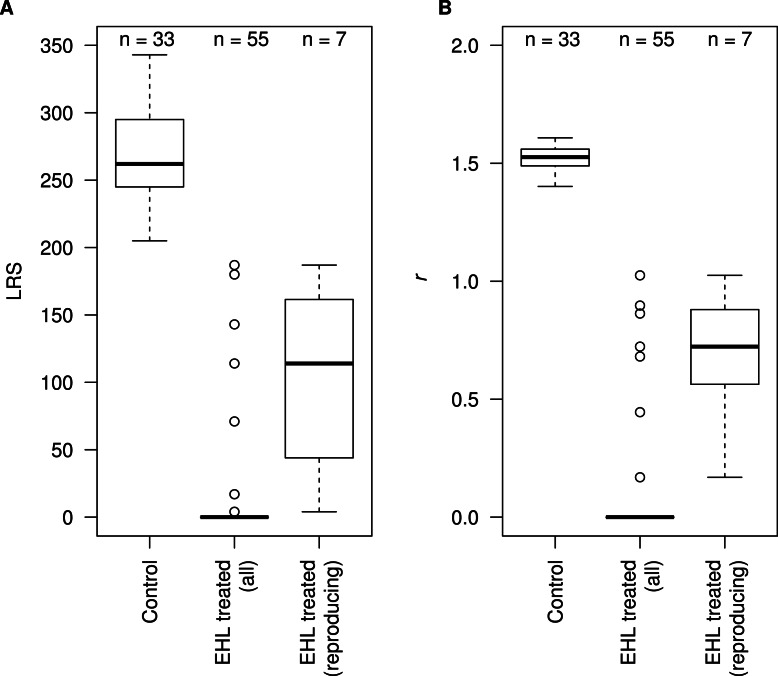
EHL reduces fecundity and slows development in *C. elegans*. Boxplots of (A) lifetime reproductive success, LRS, and (B) intrinsic rate of increase, *r*, of control worms, all EHL treated worms and of the subset of EHL treated worms that reproduced. *n* denotes the sample sizes.

To investigate these reproductive effects and the effects on survival in more detail, a larger number of worms were assayed (Fig. 5). Here, EHL treatment resulted in immediate mortality of 41% of EHL treated individuals and again EHL-treated worms were observed to remain as L1s and to arrest as dauer larvae. These results suggest that EHL treatment affects the sensory neurons. Overall, EHL treatment reduced subsequent LRS (Fig. 5A, control vs all EHL treated worms, *W* = 2,376.0, *p* < 0.001), with the subset of EHL treated worms that did reproduce producing a greatly reduced number of progeny (control vs reproducing EHL treated worms, *W* = 792.0, *p* < 0.001). A similar pattern was observed in the analysis of the effects of EHL treatment on the estimated rate of increase (Fig. 5B, control vs all EHL treated worms, *W* = 2,376.0, *p* < 0.001; control vs reproducing EHL treated worms, *W* = 792.0, *p* < 0.001).

**Figure 6:**
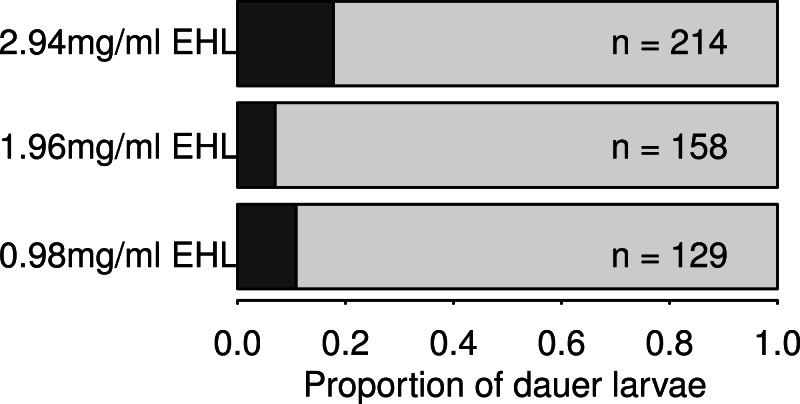
EHL treatment induces dauer larvae formation in *C. elegans.* The proportion of larvae that developed into dauer larvae observed in populations in three different EHL treatments. In the abscence of EHL treatment no dauer formation is observed under these experimental conditions in N2.

To further characterise the development of EHL-treated worms as dauer larvae, an additional set of arrested L1s were analysed. As in the assay for reproductive effects (above), some of these EHL treated worms developed as dauer larvae (Fig. 6). These worms were then SDS treated, with survival confirming that they were indeed dauer larvae. Fifty of these dauer larvae were then transferred to plates with food and maintained at 20 °C, with only one worm out of the fifty recovering and completing development as a reproductive adult after four days, and a second recovering after a total of fourteen days. Under these conditions dauer larvae normally recover rapidly and would be expected to have commenced reproduction approximately 2 days after transfer to food (Green & Harvey, 2012).

Analysis of mutant strains indicates that the ability of EHL treatment to induce dauer larvae formation varied across the genotypes (Table 1). All genotypes were however observed to show a developmental delay in response to EHL treatment, with reproduction of most EHL-treated worms not commencing until day 5 after treatment. Analysis of dauer larvae formation in PR672, *che-1(p672)* showed that this mutation does not block dauer larvae formation in response to defined amounts of pheromone and that similar numbers of dauer larvae are formed in N2 and PR672 in growing populations (*F*_1,33_ = 0.05, *p* = 0.82).

**Table 1:** Mutations affecting amphid structure can block EHL-induced dauer larvae formation. The mean dauer larvae formation found on three plates of control and 80% (1.548 mg/ml) EHL treated worms of differing genotypes of *C. elegans*.

		Mean % dauer ± S.E.		
Isolate	Genotype	Control	Lectin	Phenotype
N2	Wild-type	0	49 ± 2.5	–
CX2065	*odr-1(n1936) X*	0	53 ± 6.1	Defective chemotaxis^a^
CX2205	*odr-3(n2150) V*	0	35 ± 3.1	Defective chemotaxis^a^
				Defective osmotic avoidance^a^
PR671	*tax-2(p671) I*	0	8 ± 1.9^*^	Defective chemotaxi^b^
				Defective thermotaxis^b^
PR672	*che-1(p672) I*	0	0^*^	Defective chemotaxis^b^
PR813	*osm-5(p813) X*	0	0^*^	Defective dye filling^c^
				Defective osmotic avoidance^c^
SP1205	*dyf-1(mn335) I*	0	0^*^	Defective dye filling^d^
				Defective chemotaxis^d^
SP1709	*dyf-10(e1383) I*	0	0^*^	Defective dye filling^d^
				Defective chemotaxis^d^

**Notes.**

* Denotes genotypes where the proportion of dauer larvae observed in the EHL treatment differs from that observed in N2 (*p* < 0.05, Fisher’s Exact Test with Bonferroni adjustment to correct for multiple testing).

a Bargmann, Hartweig & Horvitz (1993).

b Dusenbery (1976).

c Dusenbery (1980).

d Starich et al. (1995).

## Discussion

We have successfully isolated the type II RIP found in the tubers of the Winter Aconite, *E. hyemalis*, by modification of a previously published protocol (Cammue, Peeters & Peumans, 1985; Kumar et al., 1993). Analysis indicates EHL is a heterodimeric protein consisting of two chains of molecular weights of approximately 28 and 31 kDa (Fig. 1). Protein sequencing confirms that EHL is a Type II RIP with the cytotoxic potential for depurination of eukaryotic ribosomes.

EHL was used to study potential lectin-mediated toxicity against *C. elegans*. The bioassays performed indicate that EHL has biocidal properties against *C. elegans*. Four phenotypic effects were identified: reduced fecundity (Figs. 4 and 5), developmental delay, chemosensory disruption and constitutive dauer formation (Fig. 6 and Table 1). *C. elegans* physiology is such that at the arrested L1 larval stage, the only cells which are not enclosed by a largely impermeable cuticle are the amphids and phasmids. These are bilaterally symmetrical sensory organs that contain the sensory neurons: each amphid containing twelve neurons and each phasmid containing two (Ward et al., 1975). Of the twelve amphid neurons, the ciliated nerve endings of eight are exposed to the external environment via the amphid pore (Ward et al., 1975). These neurons control a range of phenotypes, including egg-laying and the decision to develop as a dauer larvae (Albert, Brown & Riddle, 1981). Laser ablation of the ASI, ADF and ASG cells is sufficient to result in constitutive dauer larvae development, with ablation of the ASJ cell resulting in an inability to recover from dauer arrest (Bargmann & Horvitz, 1991). Our observations of inappropriate dauer larvae formation and the failure of most such dauer larvae to resume development indicate that EHL is interacting with these neurons and are consistent with EHL resulting in neuron death.

In general, biocidal assays with lectins involve ingestion of the lectin by the target organism. For example, EHL had previously been tested against the coleopteran pest *Diabrotica undecimpunctata howardii*, resulting in a high mortality rate and an 80% reduction in body size of survivors; there was however no previous data on reproductive effects (Kumar et al., 1993). A study of the toxic effects of the CCL2 lectin from *Coprnopsis cinerea* (Ink Cap mushroom) on *C. elegans* reported a phenotype of severe developmental delay; the lectin was adsorbed in the epithelial cells of the intestine, potentially degrading the membrane and preventing growth (Schubert et al., 2012). The absence of a food source in our assay has therefore enabled the observation of entirely new lectin-mediated *C. elegans* phenotypes induced by EHL, including a Daf-c phenotype which has not been reported before.

The cause of the developmental delay in EHL-treated worms is not clear. Possibilities would include damage to the pharynx and a subsequent reduction in pumping (feeding) ability, or, if some feeding is initiated, damage to the epithelial cells of the intestine as observed in response to the *C. cinerea* CCL2 lectin (Schubert et al., 2012). A further possibility is that differences in body size and development are also a consequence of damaged neurons (see Fujiwara, Sengupta & McIntire, 2002).

Wild-type *C. elegans* take up dyes such as DiI and FITC into the amphid neurons AWB, ASH, ASJ, ASK, ADL and ASI (Hedgecock et al., 1985). Given the likely mode of action of EHL, we reasoned that mutations that disrupt the normal formation of sensory amphids would block EHL-induced dauer larvae formation. Consistent with this, disruption of *osm-5*, *dyf-1* and *dyf-10*, all mutations in which the amphid neurons cannot take up dyes, result in no EHL-induced dauer larvae formation (Table 1). In both *odr-1* and *odr-3* mutants, where dye filling is not affected and EHL would be expected to be able to access the neurons normally, there is no reduction in dauer larvae formation in response to EHL treatment (Table 1). In contrast, dauer larvae formation is reduced in PR671, but some are still formed (Table 1), indicating that disruption of *tax-2* only partially blocks the effect. TAX-2 forms, with TAX-4, a cyclic nucleotide-gated cation channel that is required for chemotaxis in response to AWC-sensed odorants (Coburn & Bargmann, 1996). Axon outgrowth defects have however been noted in *tax-2* mutants, with c. 80% of *tax-2(p671)* animals observed to have abnormal ASJ axons (Coburn & Bargmann, 1996). It is not clear if the reduction in the EHL-induced dauer larvae formation observed in the *tax-2* mutants is a consequence of the axon guidance defects or the channel disruption.

That EHL-induced dauer larvae formation is also blocked in *che-1* mutants further supports the hypothesis that EHL is disrupting neurons. CHE-1 is a C2H2-type zinc-finger transcription factor that is required for the identity of ASE neurons (Uchida et al., 2003). Loss of CHE-1 expression eliminates the function of ASE neurons and *che-1* mutations have previously been shown to suppress Daf-c phenotypes (Reiner et al., 2008). No significant structural defects have been observed in *che-1* mutants (Lewis & Hodgkin, 1977) and our results indicate that dauer larvae formation does not appear altered in either standard dauer larvae assays or in growing populations. This thereby implies that the mutation is specifically blocking EHL-induced constitutive dauer larvae formation.

It is well established that lectins bind to glyconjugates on cell surfaces and that toxicity in RIPs is due to lectin mediated entry to the cell; this mode of action is consistent with the results presented here. In the case of *C. elegans* the only cells exposed are the amphid neurons. As a Type II RIP, EHL can be subject to retrograde transport from the cell surface along the neuronal processes, at which point the ribosomes are inactivated, causing translation to cease (Wiley, Blessing & Reis, 1982). As no post-embryonic somatic division occurs in mature individuals, and multiple chemoreceptors are expressed in a single neuron, ribosome inactivation of the neurons within the amphids would affect many functions derived from chemosensation (Sulston & Horvitz, 1977). Toxicity variables can be attributed to differing carbohydrate specificities but there is also evidence of the role of individual cell types in how they interact with lectins, indicating that any effects are characteristic of both variables (Battelli et al., 1997).

In conclusion, successful extraction using affinity chromatography has enabled assays to be conducted for biocidal properties against *C. elegans*. The results obtained demonstrate a significant reduction in fecundity, development, growth and a high incidence of abnormal dauer development when arrested L1 larvae were treated in the absence of food. The occurrence of dauer formation and a failure to recover in the presence of food supports the hypothesis that EHL is binding specifically to amphid neurons. Mutant screening has demonstrated that EHL can act as a neuronally specific cytotoxin, an effect which has previously been described with ricin and other RIPs on mammalian sensory neurons (Wiley, Blessing & Reis, 1982; Tong et al., 2012). Further studies will aim to determine if those individuals that remained as arrested L1s were doing so as a consequence of an inability to perceive the food or if an additional mechanism is at work.

Our research shows that EHL has biocidal and potential cytotoxic activity. Moreover, EHL shows specificity for GalNac, an overexpressed sugar in the Tn (GalNac clustered) antigen which characterises cancer linked O-glycans (Ju, Otto & Cummings, 2011). Other GalNac Type II RIPs such as Mistletoe Lectin (ML1) and Riproximin have demonstrated promising therapeutic relevance as anticancer agents (Voss et al., 2006; Bayer et al., 2012; Adwan et al., 2014. These factors suggest that EHL is a viable candidate for further study in respect of antineoplastic characteristics.

## Supplemental Information

10.7717/peerj.1206/supp-1 Raw data S1 Raw data for Figs. 4–6 and Table 1.
